# Morphometric MRI findings in patients with suspected autoimmune psychosis spectrum syndromes and association with EEG slowing, CSF changes, and psychometric/neuropsychological findings

**DOI:** 10.3389/fimmu.2025.1648946

**Published:** 2025-09-23

**Authors:** Katharina von Zedtwitz, Ludger Tebartz van Elst, Bernd Feige, Isabelle Matteit, Andrea Schlump, Thomas Lange, Kimon Runge, Kathrin Nickel, Nils Venhoff, Katharina Domschke, Harald Prüss, Alexander Rau, Marco Reisert, Simon J. Maier, Dominique Endres

**Affiliations:** 1 Department of Psychiatry and Psychotherapy, Medical Center - University of Freiburg, Faculty of Medicine, University of Freiburg, Freiburg, Germany; 2 Division of Medical Physics, Department of Diagnostic and Interventional Radiology, Medical Center - University of Freiburg, Faculty of Medicine, University of Freiburg, Freiburg, Germany; 3 Department of Rheumatology and Clinical Immunology, Medical Center - University of Freiburg, Faculty of Medicine, University of Freiburg, Freiburg, Germany; 4 German Center for Mental Health (DZPG), Partner Site Berlin/Potsdam, Berlin, Germany; 5 Department of Neurology and Experimental Neurology, Charité - Universitätsmedizin Berlin, Berlin, Germany; 6 German Center for Neurodegenerative Diseases (DZNE) Berlin, Berlin, Germany; 7 Department of Neuroradiology, Medical Center - University of Freiburg, Faculty of Medicine, University of Freiburg, Freiburg, Germany; 8 Department of Stereotactic and Functional Neurosurgery, Medical Center - University of Freiburg, Faculty of Medicine, University of Freiburg, Freiburg, Germany

**Keywords:** autoantibody, neuroinflammation, structural MRI, cortical thickness, brain

## Abstract

**Introduction:**

Patients with autoimmune encephalitis – who often have accompanying psychiatric symptoms – frequently have electroencephalography (EEG) changes and normal conventional magnetic resonance imaging (MRI) findings. The aim of this paper was to analyze automated EEG and morphometric MRI findings in psychiatric patients with suspected autoimmune psychosis (AP) spectrum syndromes versus controls and the correlation of MRI measures with EEG, cerebrospinal fluid (CSF), and psychometric/neuropsychological findings.

**Participants and methods:**

In total, forty patients were included. Suspected AP spectrum syndromes were defined broadly based on the autoimmune psychiatric syndrome concept. All patients showed signs of an autoimmune process. That is, upon further diagnostic testing, they tested at least positive for well-characterized neuronal antibodies, novel central nervous system antibodies, or well-characterized systemic antibodies with brain involvement. For EEG, thirty-seven matched patient-control pairs, and for structural MRI, thirty-five patients and matched controls, were available. EEG analysis for intermittent rhythmic delta/theta activity (IRDA/IRTA) was performed using independent component analysis. MRI scans were analyzed using FreeSurfer (7.2) for the subcortical measures and CAT12 for cortical thickness and global volumes.

**Results:**

Patients did not show significantly increased IRDA/IRTA rates. Regarding brain volumes, there was a significant decrease in grey matter volume/total intracranial volume (TIV) (p=0.027) and a significant increase in CSF/TIV (p=0.027), which remained significant after correction for multiple comparisons. Further differences with lower white matter volume/TIV, reduced cortical thickness in the left parahippocampal and transversotemporal gyri and an increase in the volume of the left lateral ventricle of patients did not remain significant after correcting for multiple testing. White blood cell counts in the CSF of the whole patient group correlated positively with increased hippocampal volumes. Brain volumes did not correlate with psychometric scales, but with several neuropsychological scores.

**Discussion:**

Autoantibody-associated suspected AP spectrum syndromes seem to be associated with slight global grey matter volume reductions and secondary increased CSF volumes. Associations between hippocampal volume increases and inflammatory CSF markers could, in contrast, reflect edematous swelling within the limbic system. Further multimodal imaging studies of more homogeneous AP groups might be promising to detect morphometric correlates.

## Introduction

Autoimmune psychosis (AP) could be interpreted as an oligosymptomatic subtype of autoimmune encephalitis (AE) characterized by a predominantly psychiatric presentation (1–6). AP appears to be the most common psychiatric presentation of AE (2, 5–7). Clinical manifestations might, however, go beyond classical presentations of psychosis (e.g., by manifesting as autoimmune affective, neurocognitive or obsessive-compulsive syndromes) (8–10). Therefore, some authors suggest the term autoimmune psychiatric syndrome or symptoms (APS) (6, 8, 11). Patients with AP are characterized by some specific clinical manifestations, such as acute polymorphic psychotic or catatonic syndromes (2, 7, 12). The diagnostic work-up for AP usually includes a blood test for anti-central-nervous-system (CNS) or rheumatic antibodies, electroencephalography (EEG), magnetic resonance imaging (MRI) of the brain, and cerebrospinal fluid (CSF) analysis, including routine parameters such as white blood cell (WBC) count or oligoclonal bands (OCBs) and anti-CNS antibodies (2, 7, 13, 14). CSF analyses appear to be the most sensitive tests for the detection of AP/APS (9, 10). According to the consensus criteria of Pollak et al. (2), the detection of well-characterized anti-neuronal antibodies of the immunoglobulin G (IgG) type is required for a definite AP diagnosis. In diagnostically ambiguous cases, [^18^F]fluorodeoxyglucose positron emission tomography (FDG-PET) has emerged as a valuable additional tool (6, 15–25). EEG seems to be a sensitive, albeit non-specific, diagnostic investigation for AE. Patients with NMDA-R AE, the most common form of AE with frequent additional psychotic symptoms (3, 26), show EEG pathologies in 84%–90% of cases (27, 28). In critically ill NMDA-2R AE patients in intensive care units, a specific EEG signal, the extreme delta brush, has been observed (29). In contrast, conventional MRI examinations are less sensitive and have revealed pathological findings in only approximately 33% of NMDA-R AE cases (27). Other neuronal antibodies, such as GAD65 or LGI1, are frequently associated with T2/FLAIR hyperintensities in the medial temporal lobe (1, 30, 31). Advanced quantitative MRI analyses have demonstrated structural and functional MRI changes, such as hippocampal volume loss, impaired white matter (WM) integrity, and disruption of functional brain networks, even in patients with NMDA-R AE with predominantly normal conventional MRIs (31). In psychiatric manifestations of AE, EEG abnormalities have been identified less frequently in 33%–61% of cases, and MRI alterations have been identified in 51%–57% of cases (6, 9, 10, 32). However, there has been a limited application of advanced EEG approaches—including automated analysis pipelines in which independent component analysis (ICA) is used to detect intermittent rhythmic delta and theta activity (IRDA/IRTA), as well as quantitative structural MRI analyses that use morphometric approaches—to suspected AP spectrum syndrome patient groups. A systematic literature search using the terms “(autoimmune psychosis OR autoimmune psychiatric syndrome) AND (morphometry OR morphometric OR structural MRI OR CAT12 OR FreeSurfer OR IRDA OR IRTA OR EEG slowing OR independent component analysis)” (conducted on August 4, 2025) yielded 40 hits. Among these papers, there were no comparable multimodal approaches to autoimmune-mediated psychiatric patient cohorts.

***The rationale*** of this exploratory study was to investigate EEG and morphometric MRI data in patients with suspected AP spectrum syndromes compared to healthy controls (HC). Moreover, the study sought to analyze the correlation of morphometric MRI findings with EEG slowing, CSF changes, and psychometric/neuropsychological data. Previous studies in the field of immunopsychiatry have mainly described conventional MRI findings from case series (9, 10). The present research comprises the first multimodal brain imaging study to employ automated EEG and advanced morphometric MRI analyses. From the clinical and research perspectives, such multimodal approaches are essential for an in-depth investigation of the influence of autoantibodies on brain function.

## Participants and methods

This project received approval by the Ethics Committee of the University Medical Center Freiburg (application no. EK-Freiburg: 209/18). Written informed consent was obtained from all patients and controls. This subproject was part of a larger transdiagnostic study in which multimodal brain imaging with a focus on IRDA/IRTA effects was conducted.

### Patient assessment

**Figure 1:**
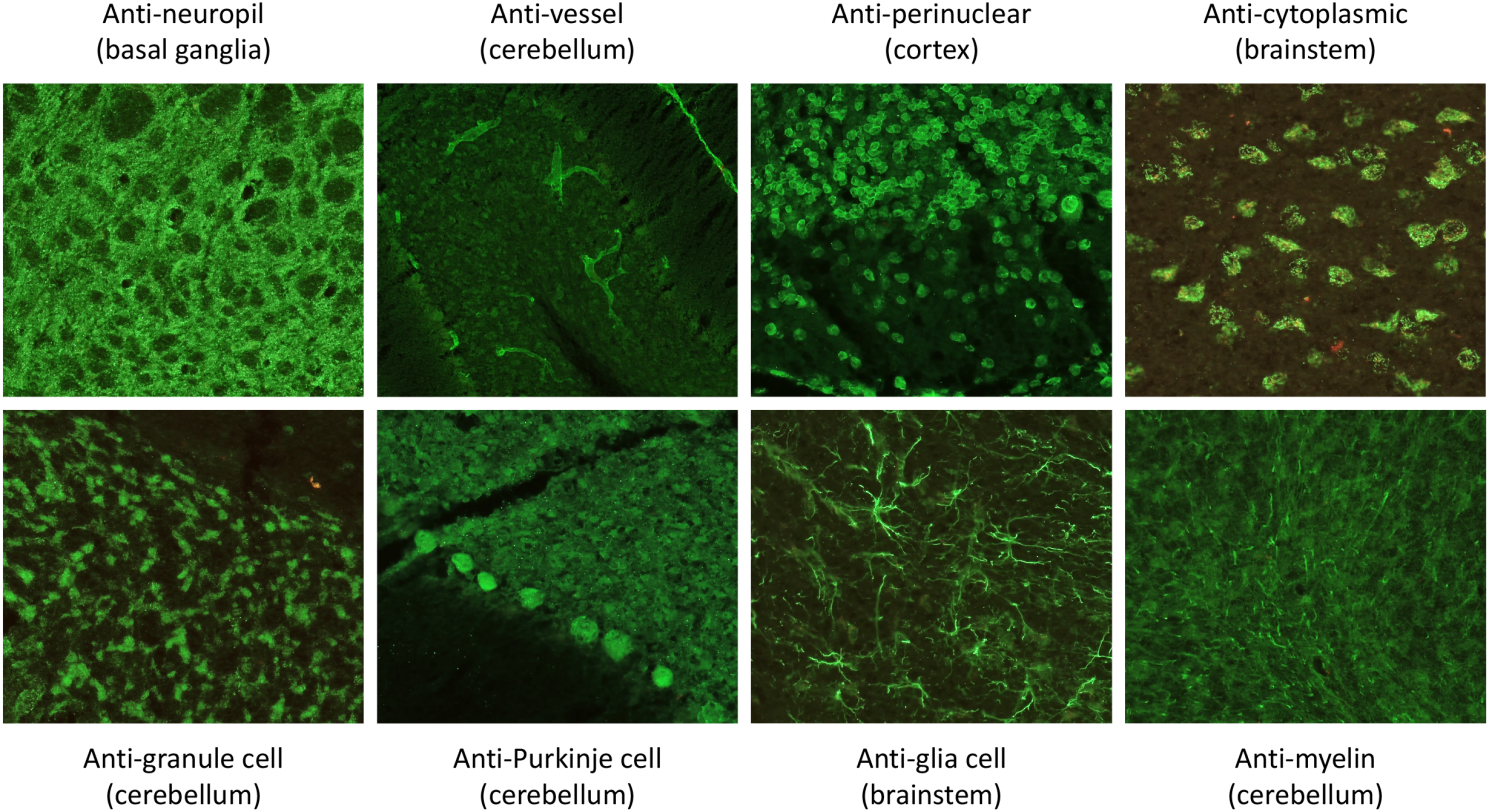
Exemplary antibody patterns using a tissue-based assay with unfixed mouse brain tissue (34), which led to inclusion in the study. This included antibody staining against the following structures: Neuropil, vessels, perinuclear and cytoplasmic structures, granule cells, Purkinje cells, glial cells, and myelin fibers. The brain region from which the antibody finding was obtained is shown in brackets. However, the staining patterns could of course also have been positive in other regions.

The suspected AP spectrum syndrome cohort was recruited from current or former patients of the Department of Psychiatry and Psychotherapy of the Medical Center at the University of Freiburg, Germany. All suitable patients whose initial diagnosis occurred within the last 10 years were offered participation in this study with further advanced MRI/EEG measurements and a broad psychometric/neuropsychological test battery. Adult patients (aged≥ 18 years) with suspected AP spectrum syndromes—characterized by predominant classical schizophreniform syndromes and predominant affective spectrum syndromes with suspected autoimmune pathophysiology—were included. All patients were positive for well-characterized neuronal antibodies (e.g., against the NMDA-R) or had novel CNS antibodies in a tissue-based assay on unfixed mouse brain slices (e.g., against vessels or granule cells; **Figure 1**). In addition, patients with well-characterized systemic antibodies (i.e., antinuclear antibodies [ANAs] measured on human embryonic kidney cells or thyroid antibodies) and clear signs of brain involvement in further diagnostic testing (i.e., who were diagnosed as neuropsychiatric lupus or Hashimoto encephalopathy; 1, 33) were included. The term suspected AP spectrum syndromes was clinically used broadly (in the current international consensus recommendations no definition of psychosis is given) (2). Antibody-associated processes do not necessarily follow the diagnostic criteria of primary psychiatric disorders. Other authors have therefore proposed the more global term APS (8, 11). This APS concept was followed here, focusing in particular on a putative common autoimmune pathomechanism – which was suspected in all patients – rather than the identical clinical phenotype. This means that not only patients with acute-onset first episode paranoid hallucinatory psychosis, but also patients with chronical symptoms and with predominant affective/neurocognitive/obsessive-compulsive syndromes were studied. If neurocognitive symptoms were present in addition to psychosis or as predominant syndrome, “predominant schizophreniform psychoses subgroup” was coded. Obsessive-compulsive syndromes in combination with affective syndromes were classified within the “predominant affective spectrum syndrome subgroup” (as was one severe obsessive-compulsive syndrome with pre-diagnosed comorbid depressive syndrome earlier in the course). Acutely ill, chronically ill and (partially) remitted patients were studied. Immunotherapy could also have been administered before the research MRI/EEG. Three subgroups according to the disease state were divided for further secondary analyses (Acute stage: current severe symptoms; Chronic stage: symptom duration exceeding 2 years; [Partially] improved: clinically documented symptom reduction following treatment). The use of psychopharmacological medication (including anticonvulsants) was recorded but did not lead to exclusion from the patient group.

As part of the diagnostic workup, patients underwent CSF analysis, conventional MRI evaluated by neuroradiology specialists, and serum and CSF antibody testing. Routine CSF parameters included WBC count, protein, albumin quotient, IgG index, and OCBs. Well-characterized neuronal antibodies against intracellular antigens were tested in serum by immunoblot (Ravo^®^, Freiburg, Germany) and against cell surface antigens in serum and CSF using a fixed, cell-based assay (Euroimmun^®^, Lübeck, Germany). In addition, most patients were tested for novel CNS antibodies using a tissue-based assay with unfixed mouse brain tissue (34). Many patients also underwent [^18^F]fluorodeoxyglucose positron emission tomography (FDG-PET; 22) as part of the clinical diagnostic work-up.

Pregnancy and lactation were defined as exclusion criteria. Subjects were also excluded if they lacked the legal capacity and ability to understand the nature, significance, and scope of the study or if they met relevant exclusion criteria for MRI examinations (pacemakers, intrauterine contraceptive devices, claustrophobia, etc.).

### Healthy control group assessment

The recruitment of the HC group was conducted via public announcements. Individuals who met the criteria for the HC group and were aged ≥18 years were included in the study. Individuals with a lifetime diagnosis of a DSM-IV axis I or II disorder (as determined by questionnaire and through objective assessment using the SCID-I screening and SCID-II testing for borderline personality disorder), as well as those currently or previously taking psychopharmacological medications or other drugs within the past six months, were excluded from participation. The use of cannabis on an episodic basis did not result in exclusion from the study. Pregnancy and lactation were defined as exclusion criteria, as well as any known relevant physical comorbidities that might influence the outcome measures. Therefore, a history of brain injury, seizures/epilepsy, (meningo)encephalitis, hydrocephalus, or space-occupying processes, was deemed grounds for exclusion. Furthermore, individuals with autoimmune diseases undergoing immunotherapy (e.g., steroids, azathioprine) or systemic autoimmune diseases with the potential for brain involvement (e.g., lupus erythematosus) were excluded from the study. Additionally, individuals were deemed ineligible if they lacked the capacity to comprehend the objectives, scope, and procedures of the study or if they met the criteria for exclusion regarding MRI (e.g., pacemaker, intrauterine contraceptive device or claustrophobia).

### Sociodemographic, psychometric and neuropsychological testing

**Figure 2:**
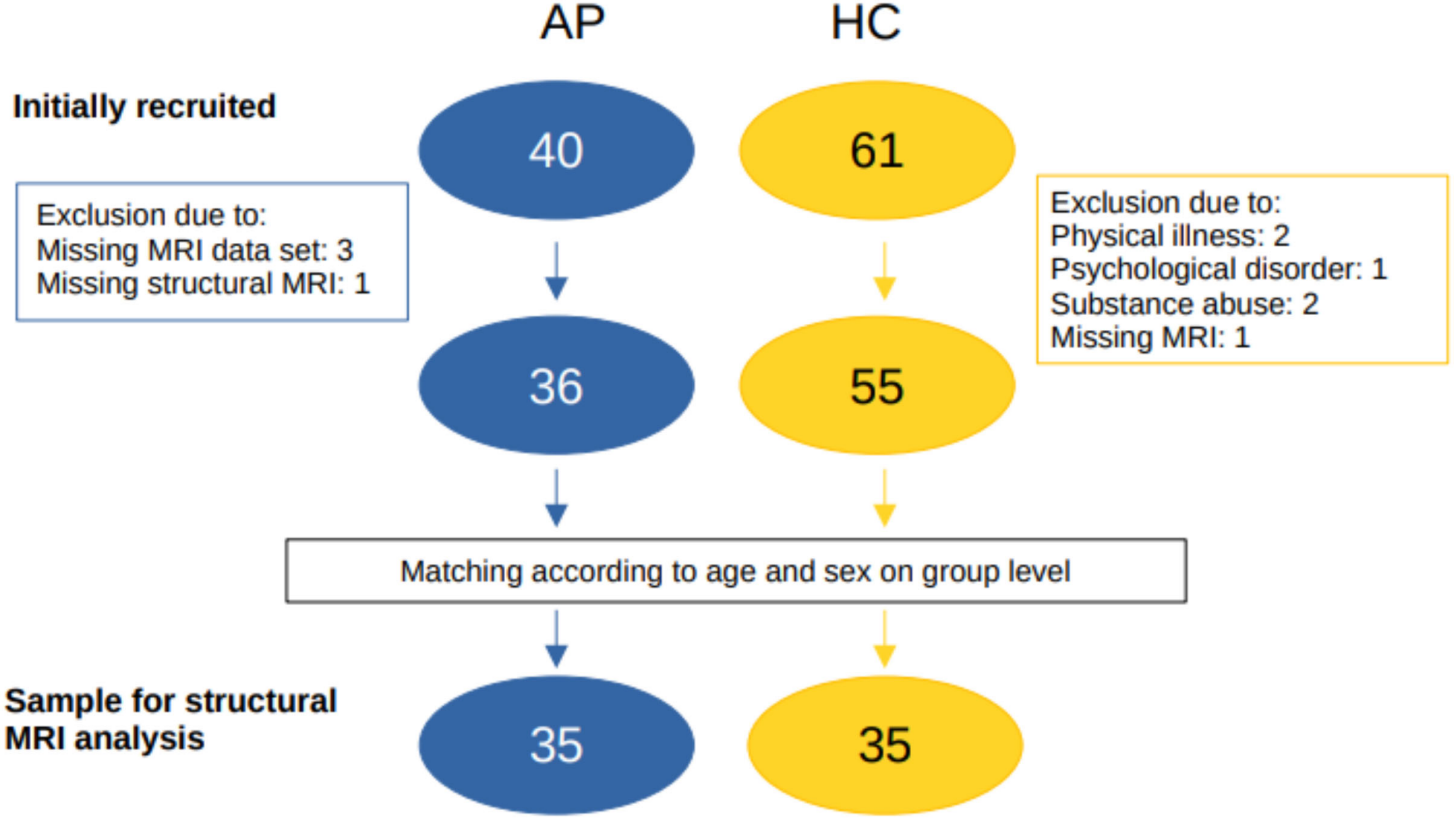
Recruitment flowchart for the group with available advanced MRI data (for the comparison of the electroencephalographic findings, 37 patients could be matched). AP, autoimmune psychosis; HC, heathy controls; MRI, magnetic resonance imaging.

The socio-demographic data were collected using a structured questionnaire. The psychometric testing included a screening using the Structured Clinical Interview for DSM-IV (SCID-I/SCID-II) (35), Eppendorfer Schizophrenia Inventory (ESI) (36), Beck Depression Inventory II (BDI-II) (37, 38), State-Trait-Anxiety-Inventory (STAI-G) (39), Symptom-Checklist (SCL-90-R) (40), Wender Utah Rating Scale (WURS) (41), ADHD-Checklist for DSM-IV (ADHD-CL) (42), Asperger Questionnaire (AQ) (43), Empathy quotient (EQ) (44), and Positive and Negative Syndrome Scale (PANSS) (45). The neuropsychological test battery consisted of the Test for Attentional Performances (TAP) (46), Verbal Learning and Memory Test (VLMT) (47), and the Culture Fair Intelligence Testing (CFT-20 R) (48). Missing questionnaires or neuropsychological test results did not result in exclusion from the study if the aforementioned inclusion criteria were met and a clear clinical diagnosis was established. Patients and controls were matched using an automatic approach on basis of age and sex on the group level. The matching process and the final study group are summarized in **Figure 2**.

### MRI measurement and analyses

The MRI measurements were all performed using the same Magnetom Prisma 3 T system (Siemens Healthineers^®^, Erlangen, Germany) at the Imaging Center of the Department of Radiology of the University Medical Center Freiburg, Germany. A 64-channel head and neck coil was used for signal reception. Morphometric analyses were based on a high-resolution T1-weighted magnetization prepared rapid gradient echo (MPRAGE) sequence with the following parameters: Field of view=256×256×160 mm^3^, voxel size=1×1×1 mm^3^; repetition time=2000 ms, echo time=4.11 ms. The Computational Anatomy Toolbox - version 12 (CAT12) was used to measure global volumes and cortical thickness (http://dbm.neuro.uni-jena.de/cat/) and subcortical analyses were based on FreeSurfer (7.2) (49; https://www.freesurfer.net/fswiki/FreeSurferWiki). CAT12 was used to process and spatially normalize T1-weighted MR images to extract global volumes and cortical thickness estimates. FreeSurfer, which includes a routine called “recon-all” that implements all the necessary steps to pre-process T1-weighted MR images, was used to extract volumes of subcortical structures (50). This combined approach using CAT12 and FreeSurfer is consistent with the conclusions of a recently published paper in which the two methods were compared (50). To further investigate the spatial pattern of AP-related changes in grey matter (GM) volume (51, 52), a voxel-based group comparisons of the CAT12-derived CSF tissue probability maps with age as nuisance covariate as implemented in the Statistical Parametric Mapping-Voxel-Based Morphometry 8-Toolbox and threshold-free cluster enhancement (53; https://github.com/markallenthornton/MatlabTFCE) was carried out. The family-wise error-method was employed to correct for multiple comparisons (i.e., across voxels).

### EEG measurement and analysis

To record the EEG measurements, a Nihon Kohden Neurofax EEG-1200 system (Nihon Kohden Corp., Tokyo, Japan) with a 10–20 electrode montage (21 Ag-AgCl sintered bridge electrodes) was used. The analog filters were set between 0.1 and 30 Hz, and the data were digitized at a rate of 200 Hz. Electrode impedances were kept below 5kΩ. EEG recording lasted for 11 minutes and included eye-opening and -closing maneuvers and a three-minute hyperventilation (HV) episode. No photostimulation was performed. The IRDA/IRTA rates were determined after using ICA to separate eye movement artifacts as well as possibly multiple IRDA/IRTA components with different origins, using in-house software (https://github.com/berndf/avg_q, 54). To begin this process, artifacts were detected by identifying large amplitudes, large derivatives (“jumps”), and successive points with negligible variation (“blocking”). The five seconds before and after any artifact or EEG start/stop event were marked for exclusion from the analysis. The remaining artifact-free EEG sections were then used for ICA training. Detection of IRDAs/IRTAs in the non-artifact ICA components were counted if they exceeded a lenient amplitude threshold of 1 µV and occurred in the artifact-free sections. Finally, the number of IRDA/IRTA events per minute of artifact-free EEG was computed (i.e., the sum of IRDA and IRTA events, not the ratio between them). Notably, this method extracted IRDA/IRTA activity regardless of the location of its generators. IRDA/IRTA rates were calculated separately for the EEG sections before and after HV. The rate before HV and the difference after-before HV (IRDA difference) are then analyzed statistically (54, 55).

### Statistical analyses

Statistical analyses were conducted using R software 3.6.0 (R software v.3.6.0, R Foundation for Statistical Computing Platform, Vienna, Austria). Group comparisons for categorical variables (e.g., sex) were carried out with Fisher’s exact test. A t-test for independent samples was used for dimensional clinical variables (e.g., age). Based on prior studies of NMDA-R encephalitis (26), it was assumed before the study began that our patient cohort would exhibit consistent clinical EEG abnormalities. However, this did not turn out to be true. The Shapiro–Wilk test was used to test for normal distribution. Automatically analyzed EEG slowing, meaning IRDA/IRTA rates, was compared using a Wilcoxon rank sum test. All thickness and volume measures were adjusted for the effects of age (linear and quadratic terms), sex, and image quality rating (IQR) using a linear model and the predict function in R, standardizing the data to the mean age and mean IQR value of the sample. The “predict” function was used in two ways: first, without specifying new data, to obtain each subject’s fitted value from the model, which was then subtracted from the observed value; and second, with new data corresponding to target covariate levels (the sample’s mean age, IQR, predefined sex, and total intracranial volume [TIV]) to obtain the model’s predicted value at those reference levels. The adjusted value was computed as the observed value minus the fitted value plus the prediction at the reference covariate levels. A robust two-sample trimmed t-test for unequal population variances was used for group comparisons of morphometric brain imaging data (56), as the data were predominantly not normally distributed. In addition, effect sizes (Cohen’s D) were calculated using R (57–59). Correlation analyses were performed using the *pbcor* function from the *WRS2* package in R to compute the percentage bend correlation coefficient. For the correlation with the neuropsychological findings, age adjusted neuropsychological raw data were used. Therefore, all raw TAP subtest scores were screened for potential age and sex influences with the Boruta feature-selection wrapper for the random forest algorithm; age emerged as an important predictor, while sex did not. Consequently, each raw score was regressed on age (lm[raw ~ age]), and an age-adjusted value for each participant was calculated as the residual plus the model prediction at the sample’s mean age, similar to the “predict” approach mentioned above. These age-corrected scores replaced the original t-scores and were used in subsequent analyses. The corrections for multiple testing were performed for all tests according to the Benjamini–Hochberg approach (60). A significance level of p<0.05 was applied. All corrected p-values are described as adjusted (“p_adj_”) to distinguish them from the uncorrected p-values (“p”) that are also reported.

## Results

### Patient and control group

A total of 35 patients with MRI data (mean age: 41 ± 15 years; 51% female) were included, alongside 35 age- and sex-matched HCs. No significant differences were identified in age (p=0.06) or sex (p=0.80) compared to the HC group. The patient sample was almost evenly split between predominant classical schizophreniform syndromes in 51% and predominant affective spectrum syndromes in 49%. Overall, eleven patients (31%) had well-characterized antibodies and 24 patients (69%) novel anti-CNS antibodies in serum and/or CSF. Antibody testing in CSF was positive in 77% (in 27 from 35 cases) of the entire patient group. Clinical routine MRI alterations were detected in 80% (without non-specific white matter changes or other norm variants in only 29%), CSF routine pathologies in 51%, FDG-PET (only available in n=23) hyper/hypometabolism in 52%, and clinical EEG alterations in 37%. The detailed clinical syndromes, diagnostic findings, including different autoantibodies and medications, are summarized in **Table 1**. Only 29% of the included patients were in an acute stage, of which five patients had already received immunotherapy. In total, twenty-two patients (63%) received immunotherapies before MRI scanning (no acute immunotherapies were administered during the time of MRI). While the HC group received no medication, suspected AP spectrum syndrome patients were most likely to receive additional antipsychotics (60%) and antidepressants (40%). On the psychometric PANSS, ESI, BDI-II, AQ, ADHD-CL, SCL-90-R, and STAI-G, the patient group scored significantly higher. For the neuropsychological testing including TAP, the VLMT, and the CFT20-R various significant differences with generally more pathological scores in the patient group in contrast to HCs have been detected (**Tables 2**, **3**).

**Table 1:** Detailed presentation of the detected antibody and clinical findings in patients with suspected autoimmune psychosis spectrum syndromes.

Suspected AP spectrum syndromes (N=35)	
Clinical routine findings	
Well-characterized anti-CNS or systemic antibodies* In serum overall Anti-LGI1 Anti-NMDA-R Anti-MOG Anti-Yo Anti-CASPR2 Anti-VGCC Anti-TPO ANAs **In CSF overall** Anti-LGI1 Anti-NMDA-R Anti-MOG Anti-Yo Anti-CASPR2 Anti-VGCC Anti-TPO ANAs	11 (31%) 1 (2.9%) 1 (2.9%) 2 (5.7%) 1 (2.9%) 1 (2.9%) 1 (2.9%) 2 (5.7%) 1 (2.9%) 1 (2.9%) 2 (5.7%) 0 (0%) 0 (0%) 0 (0%) 0 (0%) 0 (0%) 1 (2.9%)
**Novel anti-CNS antibodies** Positive tissue-based assay in serum and/or CSF overall* In serum overall Anti-vessel pattern Anti-granule cell pattern Anti-myelin pattern Anti-Purkinje cell pattern Anti-cytoplasmic pattern Anti-glia cell pattern Anti-axon initial segment pattern Anti-neuropil pattern Anti-perinuclear pattern Anti-hippocampal pattern **In CSF overall** Anti-vessel pattern Anti-granule cell pattern Anti-myelin pattern Anti-Purkinje cell pattern Anti-cytoplasmic pattern Anti-glia cell pattern Anti-axon initial segment pattern Anti-neuropil pattern Anti-perinuclear pattern Anti-hippocampal pattern	24 (69%) 2 (5.7%) 2 (5.7%) 3 (8.6%) 0 (0%) 2 (5.7%) 0 (0%) 1 (2.9%) 1 (2.9%) 2 (5.7%) 0 (0%) 5 (14%) 4 (11%) 3 (8.6%) 2 (5.7%) 1 (2.9%) 1 (2.9%) 1 (2.9%) 1 (2.9%) 3 (8.6%) 2 (5.7%)
**Syndrome overall ** Predominant schizophreniform syndromes** Predominant affective spectrum syndromes***	18 (51%) 17 (49%)
**Duration of illness ** Acute stage**** Chronic stage (> 2 years) (Partially) improved by treatment	10 (29%) 11 (31%) 14 (40%)
**Current psychiatric medication ** **(< 6 month)** Atypical antipsychotics Typical antipsychotics Antipsychotics Antidepressants Mood stabilizers Anticonvulsants Benzodiazepines	20 (57%) 5 (14%) 21 (60%) 14 (40%) 6 (17%) 5 (14%) 4 (11%)
**Previous psychiatric medication ** **(> 6 month) ** Atypical antipsychotics Typical antipsychotics Antidepressants Mood stabilizers Anticonvulsants Benzodiazepines	16 (47%) 2 (5.9%) 21 (62%) 2 (5.9%) 9 (26%) 8 (24%)
**CSF findings (N=35) ** White blood cell count (/µl) - Increased Protein concentration (mg/l) (N=34) - Increased Albumin quotient - Increased IgG-Index - Increased OCBs in serum OCBs in CSF **CSF abnormalities**	2.20 ± 1.92 4 (11%) 439 ± 214 12 (34%) 6.57 ± 4.33 11 (31%) 0.54 ± 0.16 2 (5.7%) 0 (0%) 4 (12%) **18 (51%)**
**Clinical EEG pathologies (N=35) ** Focal slowing Generalized slowing Intermittent slowing Continuous slowing Spike wave activity **Overall EEG alterations**	1 (4%) 12 (34%) 11 (31%) 1 (3%) 0 (0%) **13 (37%)**
Clinical routine brain imaging findings	
**Clinical routine MRI changes (N=35) ** Non-specific white matter changes (Chronic) inflammatory lesions Global atrophy Focal atrophy Pineal cysts Others **Overall MRI alterations**	26 (74%) 3 (9%) 6 (17%) 5 (14%) 6 (17%) 6 (17%) **28 (80%)**
**FDG-PET alterations (N=23) ** Hypermetabolism Hypometabolism **Overall FDG-PET alterations**	6 (26%) 8 (35%) **12 (52%)**

*Only the predominant antibodies were mentioned (in the event that several antibodies were present). **Three patients had additional severe neurocognitive syndromes, in one further patient the predominant neurocognitive syndrome was the leading clinical syndrome (but the patient was pre-diagnosed with psychosis) ***Seven patients had additional obsessive-compulsive syndromes and one patient had a leading severe obsessive-compulsive-syndrome (without current affective syndrome, but with pre-diagnosed additional depression) ****Five cases received immunotherapy. AP, Autoimmune psychosis; CSF, Cerebrospinal fluid; CNS, central nervous system; EEG, Electroencephalography; FDG-PET, Fluorodeoxyglucose positron emission tomography; IgG, Immunoglobulin G; MRI, Magnetic resonance imaging; OCB, oligoclonal bands.

**Table 2:** Clinical and psychometric characteristics of the study cohort and comparison of patients with suspected autoimmune psychosis (AP) spectrum syndrome and healthy control (HC) group.

	Suspected AP spectrum syndromes (N=35)^1^	HC (N=35)^1^	p-value^2^
Clinical data			
**Age in years**	41 ± 15 (N=35)	35 ± 11 (N=35)	0.058
**Sex ** female (%) male (%)	18 (51%) (N=35) 17 (49%) (N=35)	20 (57%) (N=35) 15 (43%) (N=35)	0.800
**Body information ** BMI (kg/m^2^) Handedness - both - left - right	25.8 ± 4.2 (N=35) 2 (7%) (N=29) 2 (7%) (N=29) 25 (71%) (N=29)	22.6 ± 2.5 (N=35) 1 (2.9%) (N=35) 4 (11%) (N=35) 30 (86%) (N=35)	**<0.001** 0.600
**Mother tongue ** German Chinese Romanian	34 (97%) (N=35) 0 (0%) (N=35) 1 (3%) (N=35)	33 (94%) (N=35) 1 (3%) (N=35) 1 (3%) (N=35)	>0.900
**Academic degree ** University degree High degree Middle degree Low degree Other qualification	8 (23%) (N=35) 9 (26%) (N=35) 11 (31%) (N=35) 2 (6%) (N=35) 5 (14%) (N=35)	23 (66%) (N=35) 8 (23%) (N=35) 4 (11%) (N=35) 0 (0%) (N=35) 0 (0%) (N=35)	**0.001**
**Current employment status ** Full-time job Part-time job Student Trainee Pensioner Unemployed	2 (6%) (N=35) 8 (23%) (N=35) 4 (11%) (N=35) 1 (3%) (N=35) 13 (37%) (N=35) 7 (20%) (N=35)	15 (43%) (N=35) 9 (26%) (N=35) 8 (23%) (N=35) 2 (6%) (N=35) 0 (0%) (N=35) 1 (3%) (N=35)	**0.001**
Psychometric scores			
Questionnaire	p-value^2^	Questionnaire	p-value^2^
**PANNS ** PANSS sum (33/35) PANSS positive (34/35) PANSS negative (33/35) PANSS general (34/35)	**<0.001** **<0.001** **<0.001** **<0.001**	**ESI** ESI attention and speech impairment (30/34) ESI auditory uncertainty (31/34) ESI deviant perception (31/34) ESI ideas of reference (30/33) ESI frankness (30/34)	**0.001** **0.023** **0.035** **0.029** >0.900
**BDI-II (32/35)** **EQ (32/35)** **AQ (32/35)** **WURS (29/35)** **ADHD-Checklist (28/34)** **STAI-G Trait (32/35)**	**<0.001** 0.4 **<0.001** 0.3 **<0.001** **<0.001**	**SCL-90-R (32/35)** Hostility Anxiety Depression Somatization Obsessive-compulsive Interpersonal sensitivity Phobic anxiety Paranoid ideation Psychoticism	**0.007** **<0.001** **<0.001** **0.004** **<0.001** **0.006** **0.002** **0.012** **0.002**

^1^Mean ± SD; n (%). ^2^Welch Two Sample t-test or Fisher’s exact test or Wilcoxon rank sum test. ADHD, Attention deficit hyperactivity disorder; AP, Autoimmune psychosis; AQ, Autism Quotient; BDI-II, Beck Depression Inventory II; BMI, Body Mass Index; ESI, Eppendorfer Schizophrenia Inventory; EQ, Emotional quotient; HC, Healthy controls; PANNS, Positive and Negative Syndrome Scale; SCL-90R, Symptom Checklist-90-R; STAI-G, State-Trait Anxiety Inventory; WURS, Wender Utah Rating Scale.

Significant group differences are marked in bold.

**Table 3:** Neuropsychological characteristics of the study cohort and comparison of patients with suspected autoimmune psychosis (AP) spectrum syndrome and healthy control (HC) group.

Test	p-value^1^	Test	p-value^1^
Neuropsychological findings			
**TAP ** Alertness no warning tone (28/34) Alertness warning tone (28/34) Phasic alertness (28/34) Working memory mistakes (28/34) Working memory missings (28/34) Mental flexibility (27/34) Divided attention mistakes (27/34) Divided attention missings (27/34)	**0.034** 0.081 0.088 **0.007** **0.001** **0.001** **0.036** **0.003**	**VLMT** VLMT learning (35/35) VLMT false positive (34/35) VLMT preservations (34/35) VLMT recognition (33/35) VLMT Consolidation (33/35)	**<0.001** 0.095 0.274 **<0.001** **<0.001**
**CFTR-20 IQ (24/31)**	**<0.001**		

^1^Welch Two Sample t-test or Fisher’s exact test or Wilcoxon rank sum test. AP, Autoimmune psychosis; CFTR-20, Culture Fair Intelligence Testing; HC, Healthy controls; TAP, Test of Attentional Performance; VLMT, Verbaler Lern- and Merkfaehigkeitstest.

Significant group differences are marked in bold.

### Electroencephalography findings

**Figure 3:**
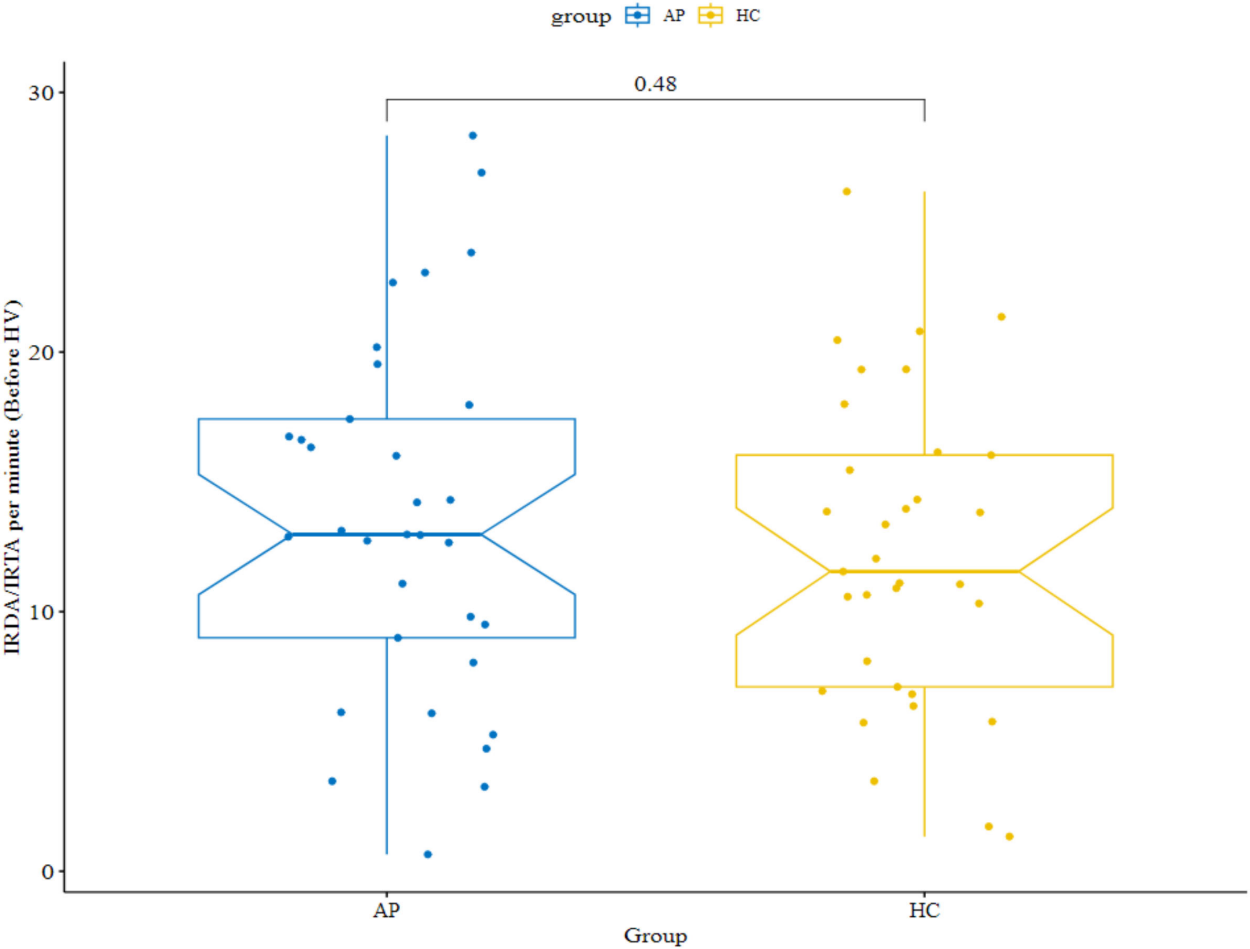
Intermittent rhythmic delta/theta activity (IRDA/IRTA) rates by group before hyperventilation (HV). The results of the 35 patients and 35 controls from whom imaging data was also available are shown. AP, autoimmune psychosis; IRDA/IRTA, intermittent rhythmic delta/theta activity; HC, healthy controls; HV, hyperventilation.

Among the 35 patients with available MRI data and their matched controls, IRDA/IRTA rates were not significantly different, i.e., events per minute before HV (14.0 ± 7.0 in AP vs. 12.0 ± 6.0 in HC; p=0.483), and the IRDA/IRTA difference (1.8 ± 4.8 in AP vs. 3.1 ± 4.3 in HC; p=0.194) (**Figure 3**). In the slightly larger EEG subset of 37 patient-control pairs (including those without MRI data), the negative findings with IRDA/IRTA rates per minute before HV (13.6 ± 6.0 in AP vs. 11.9 ± 5.9 in HC; p=0.220) and IRDA/IRTA difference (1.9 ± 4.7 in AP vs. 3.4 ± 4.2 in HC; p=0.102) remained consistent.

### Global magnetic resonance imaging volumes

**Figure 4:**
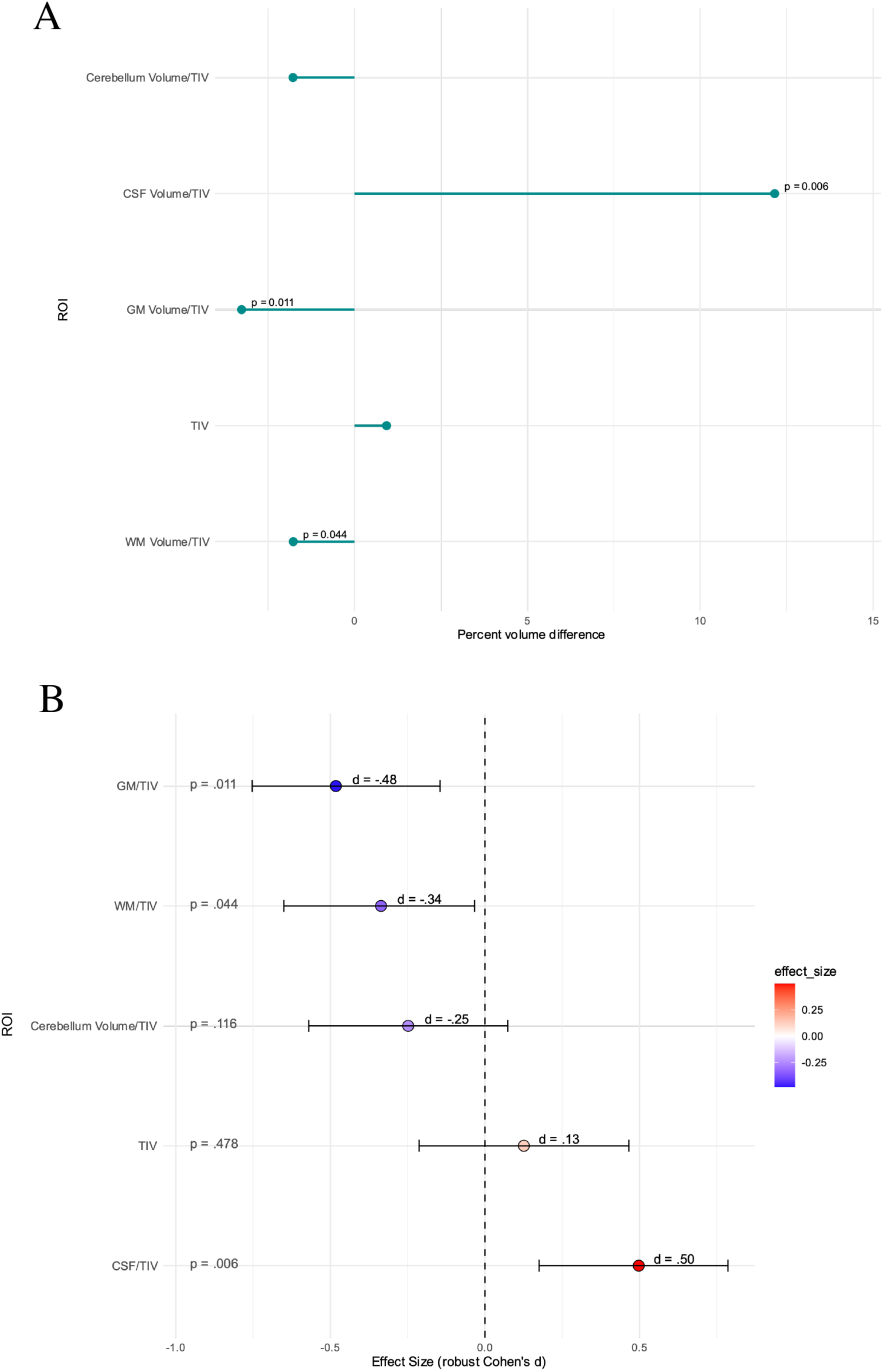
Differences in global volumes between healthy controls (HC) and suspected autoimmune psychosis (AP) spectrum syndromes. **(A)** In percent. **(B)** With effect size confidence intervals. Presented are p-values before correction for multiple comparisons, after correction for multiple testing only the ratios of CSF/TIV (p_adj_=0.027) and GM/TIV (p_adj_=0.027) remained significant, WM/TIV still showed a trend towards reduced volumes in the patient group (p_adj_=0.073). AP, autoimmune psychosis; CSF, cerebrospinal fluid; GM, grey matter; TIV, total intracranial volume; WM, white matter.

The analysis of global brain volumes showed increased ratios of CSF/total intracranial volume (TIV) (p=0.006) and reduced ratios of GM/TIV and WM/TIV in the patient group (p=0.011 and p=0.044, respectively). TIV did not differ significantly between groups, and cerebellar volume also showed no significant group differences (**Figure 4**). After correction for multiple testing, only the ratios of CSF/TIV (p_adj_=0.027) and GM/TIV (p_adj_=0.027) remained significant, WM/TIV still showed a trend towards reduced volumes in the patient group (p_adj_=0.073).

### Cortical thickness

**Figure 5:**
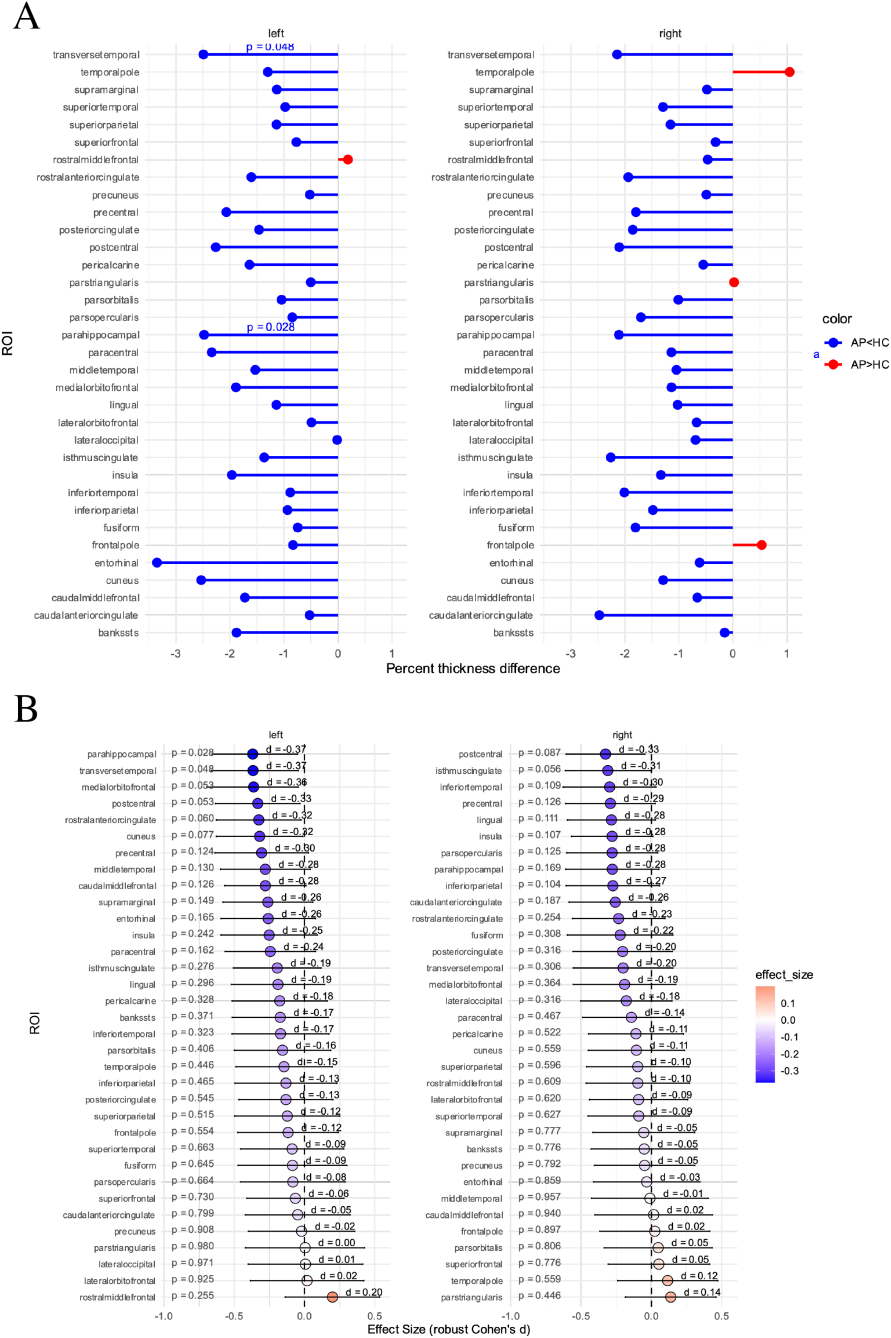
Differences in cortical thickness between healthy controls (HC) and suspected autoimmune psychosis (AP) spectrum syndromes. **(A)** In percent. **(B)** Effect size with confidence intervals. Presented are p-values before correction for multiple comparisons, after correction for multiple testing no significant differences remained (left parahippocampal gyrus: p_adj_=0.520; left transversotemporal gyrus: p_adj_=0.520). AP, autoimmune psychosis; HC, healthy controls; ROI, region of interest.

The analysis of cortical thickness identified widespread reductions with small effect sizes across almost all analyzed regions in the suspected AP spectrum group. Significant reductions were observed in the left parahippocampal gyrus (p=0.028) and the left transversotemporal gyrus (p=0.048) (**Figure 5**). However, these effects did not withstand the correction for multiple testing (left parahippocampal gyrus: p_adj_=0.520; left transversotemporal gyrus: p_adj_=0.520).

### Subcortical volumes

**Figure 6:**
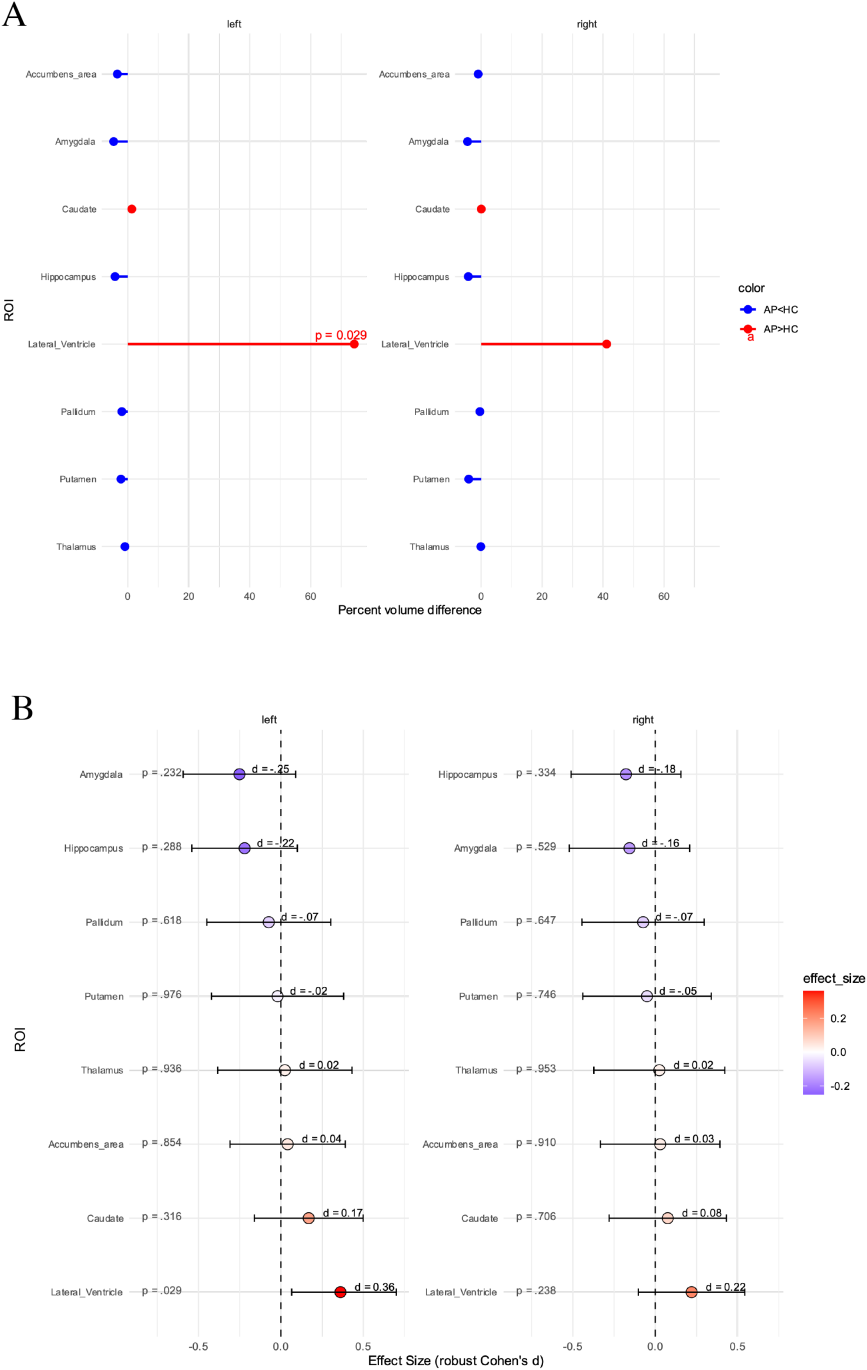
Differences in subcortical volume between healthy controls (HC) and suspected autoimmune psychosis (AP) spectrum syndromes. **(A)** In percent. **(B)** Effect size with confidence intervals. Presented are p-values before correction for multiple comparisons, after correction for multiple testing the differences for the left lateral ventricle no longer remained significant (p_adj_=0.461). AP, autoimmune psychosis; HC, healthy controls; ROI, region of interest.

**Figure 7:**
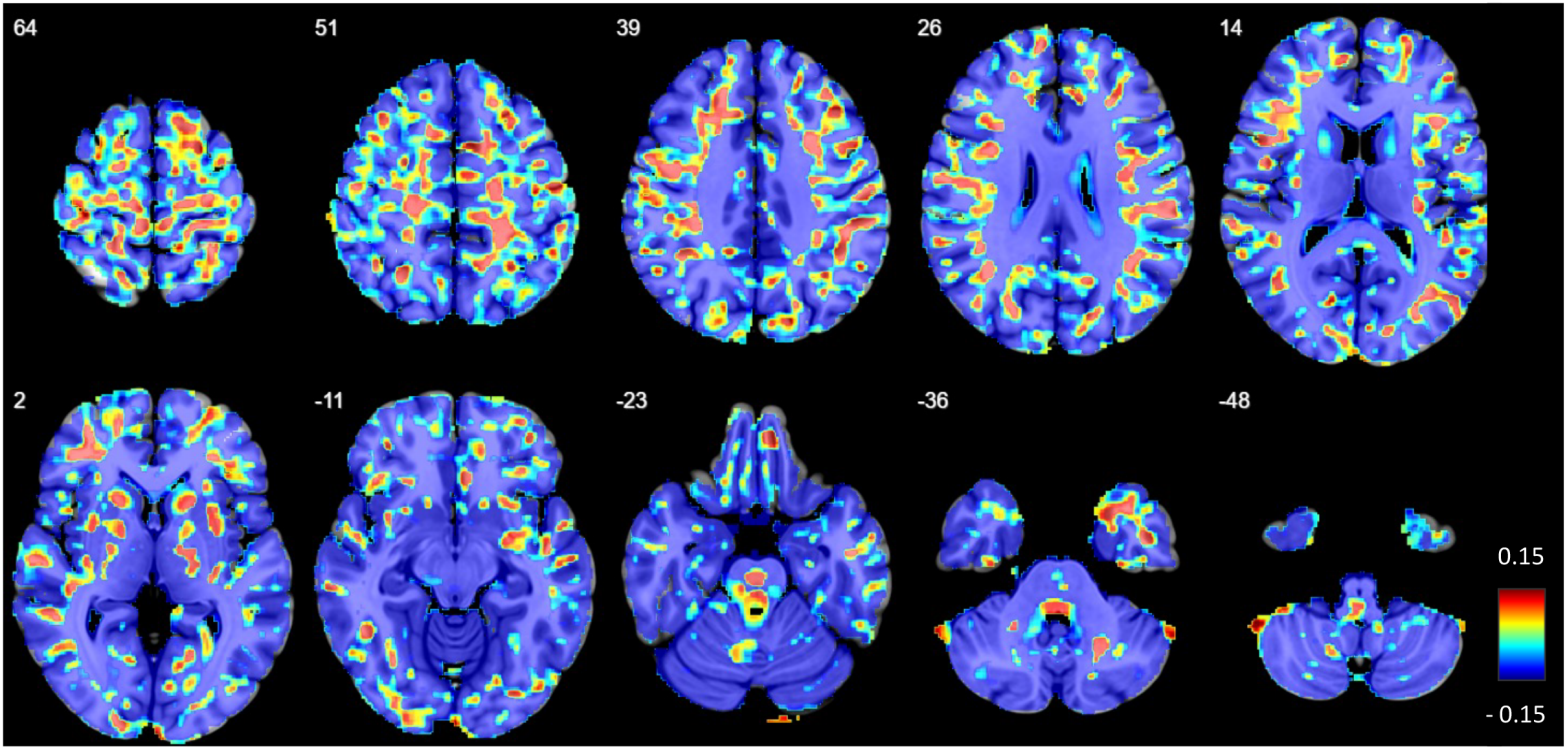
The standardized regression coefficients of the factor cerebrospinal fluid (CSF) were extracted from two-tailed linear regression models obtained from voxel-wise comparisons between suspected autoimmune psychosis (AP) spectrum syndromes and healthy controls (HC) and superimposed on a T1w magnetic resonance imaging (MRI) template. Color coding indicates coefficient values as a measure of the effect size of the AP factor (hot colors: positive effects vs. cold colors: negative effects).

The analysis of subcortical volumes revealed no significant differences, except for the left lateral ventricle, which was significantly enlarged in the patient group (p=0.028); however, the finding was not significant after correction for multiple testing (p_adj_=0.461) (**Figures 6**, **7**).

### Subgroup analyses according to disease state

The different findings regarding global brain volumes, cortical thickness, and subcortical volumes (including those that were significant before correction for multiple testing) between the patient and the HC group were more precisely analyzed in a subgroup analysis of the suspected AP group with regard to disease state. Specifically, patients in the acute stage (N=10) were compared with those in the chronic stage (N=11) and those in the non-acute stage (chronic plus [partially] improved, N=25). No significant differences were found for CSF/TIV (p=0.642, p=0.522), GM/TIV (p=0.637, p=0.589), WM/TIV (p=0.646, p=0.518) ratios, cortical thickness of the left parahippocampal (p=0.890, p=0.767) and left transversotemporal (p=0.578, p=0.454) gyri, or the volume of the left lateral ventricle in either the acute vs. chronic or acute vs. non-acute comparisons (p=0.253, p=0.094).

### Correlation analyses within the patient group

#### Electroencephalography

**Figure 8:**
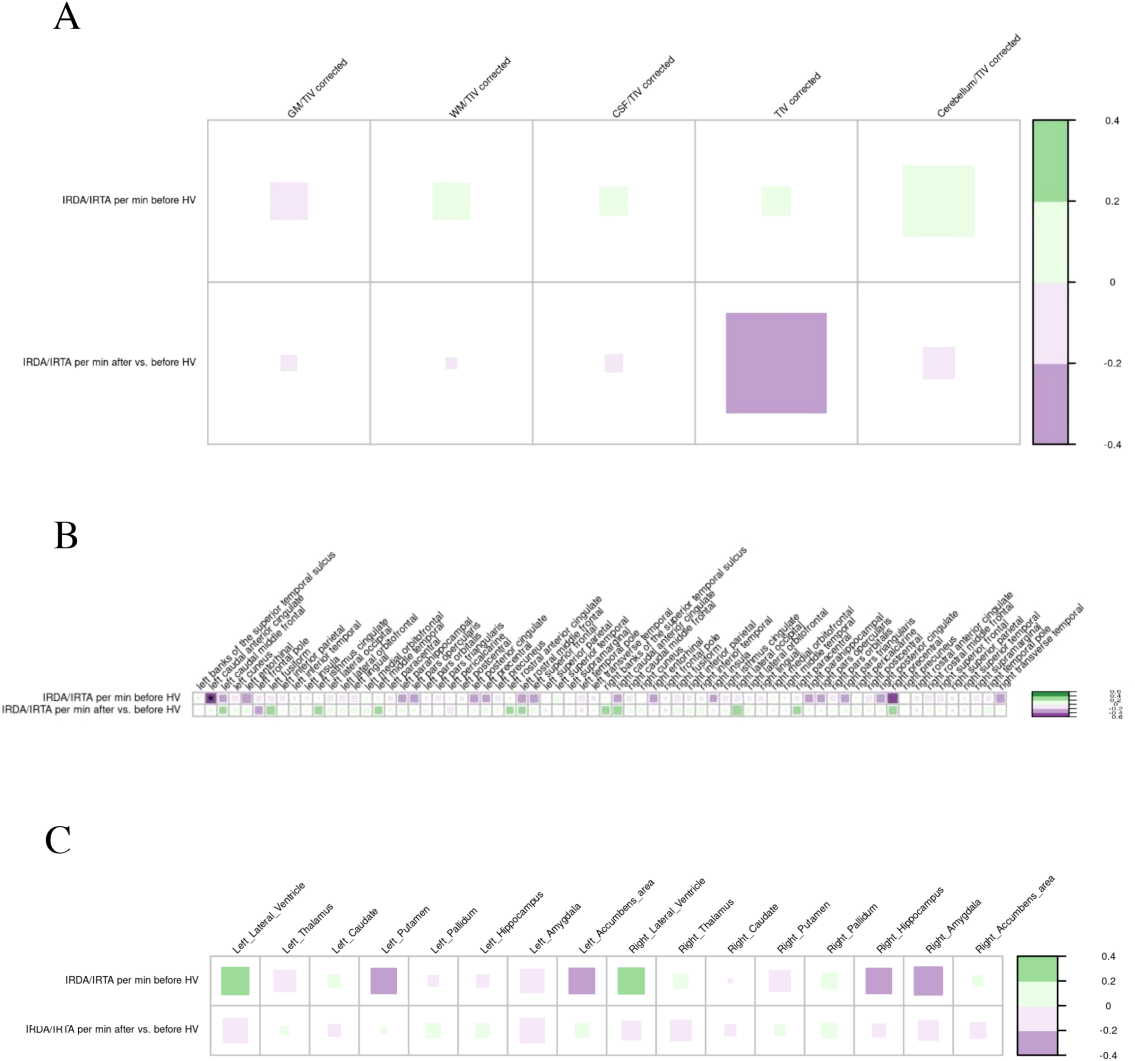
Correlation of the electroencephalography slowing parameters with global volumes **(A)**, cortical thickness **(B)**, and subcortical volumes **(C)** in the suspected autoimmune psychosis spectrum syndromes. The larger and more colorful the boxes are, the closer the described correlation is to significance. If an asterisk (*/**/***) appears, significance is achieved: *p-value < 0.05; **p-value < 0.01; ***p-value < 0.001. CSF, cerebrospinal fluid; GM, grey matter; IRDA/IRTA, intermittent rhythmic delta/theta activity; HV, hyperventilation; min, minute; TIV, total intracranial volume; WM, white matter.

No significant correlations were identified between EEG slowing parameters and global MRI volumes. However, a higher IRDA/IRTA rate per minute before HV in the EEG was associated with cortical thinning of the left caudal anterior cingulate cortex (p<0.05). The correlation of IRDA/IRTA rates with subcortical volumes revealed no significant findings (**Figure 8**).

#### Cerebrospinal fluid

**Figure 9:**
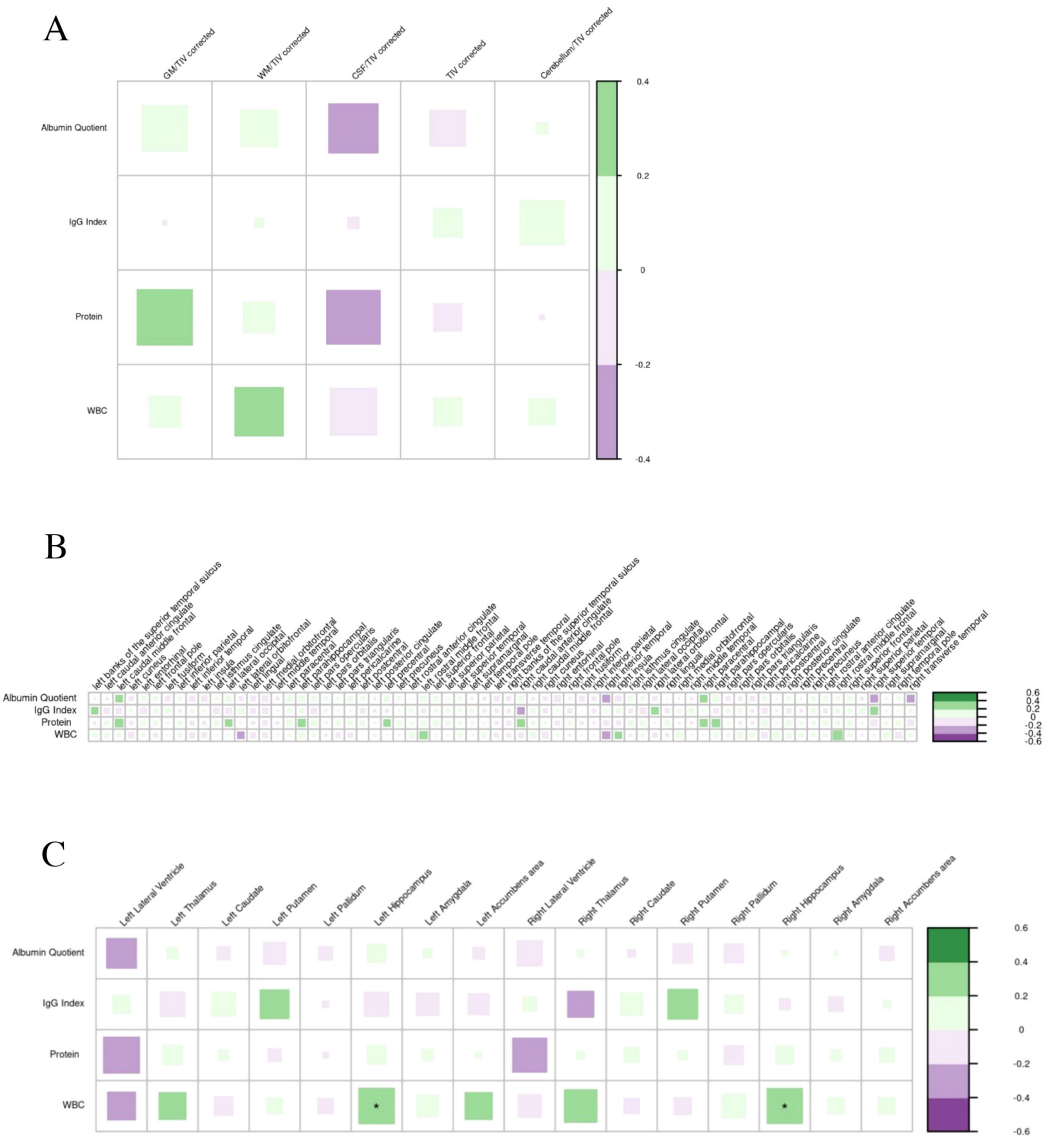
Correlations of cerebrospinal fluid findings (only dimensional parameters were analyzed) with global volumes **(A)**, cortical thickness **(B)**, and subcortical volumes **(C)** in suspected autoimmune psychosis spectrum syndromes. The larger and more colorful the boxes are, the closer the described correlation is to significance. If an asterisk (*/**/***) appears, significance is achieved: *p-value < 0.05; **p-value < 0.01; ***p-value < 0.001. CSF, cerebrospinal fluid; GM, grey matter; IgG, immunoglobulin G; TIV, total intracranial volume; WBC, white blood cell; WM, white matter.

Global MRI volumes and cortical thickness showed no significant associations with CSF findings. However, WBC count in CSF was positively correlated with larger volumes in the left and right hippocampus (p<0.05; **Figure 9**). An additional comparison of the WBC counts between the acutely ill and the non-acutely (p=0.769) or chronically ill (p= 0.705) showed no significant group difference in the WBC counts. No significant correlation was identified between the advanced MRI parameters and the CSF markers for blood-brain barrier function (albumin quotient and protein concentration).

#### Psychometric findings

**Figure 10:**
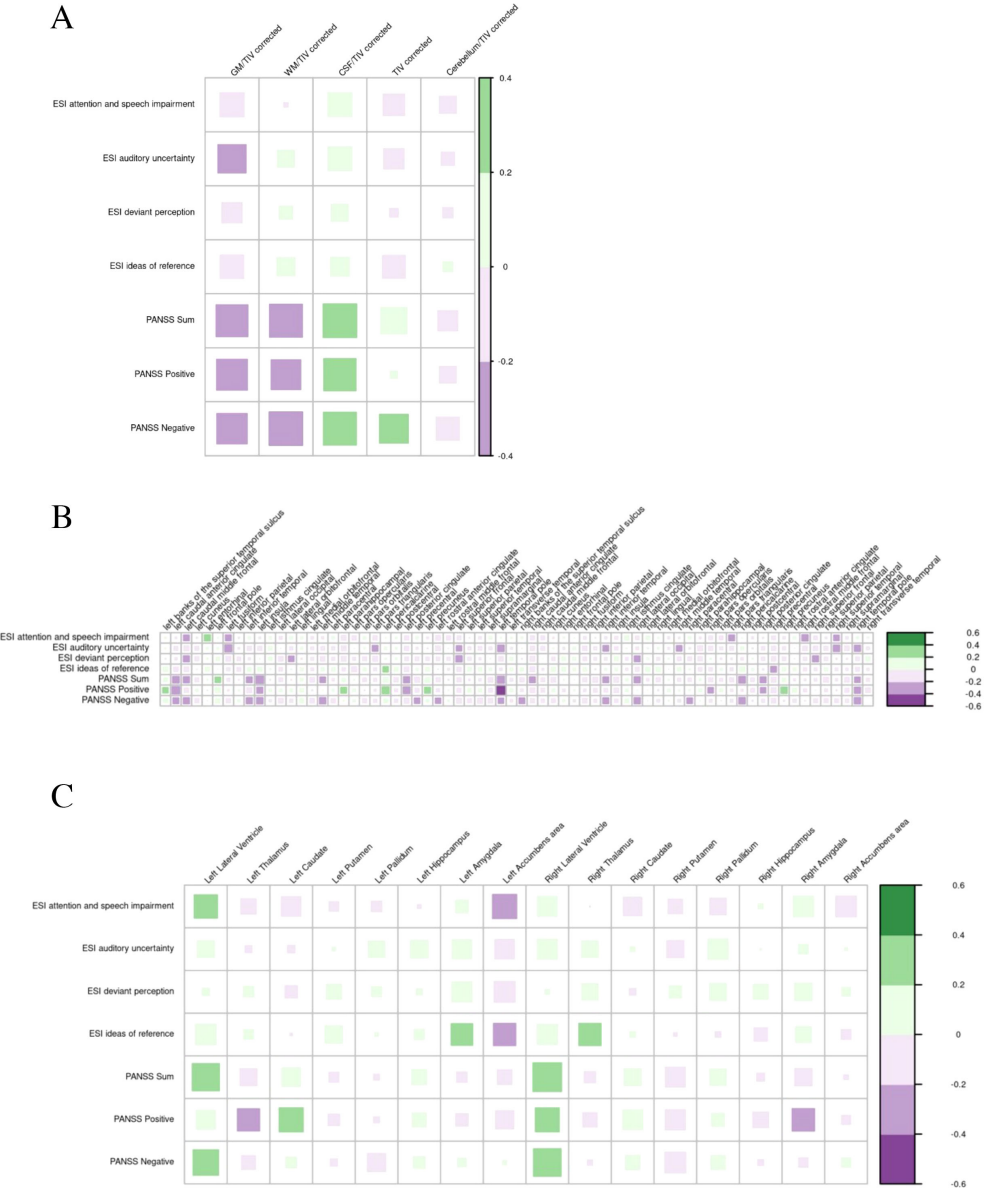
Correlations of psychometry with global volumes **(A)**, cortical thickness **(B)**, and subcortical volumes **(C)** in suspected autoimmune psychosis spectrum syndromes. The larger and more colorful the boxes are, the closer the described correlation is to significance. If an asterisk (*/**/***) appears, significance is achieved: *p-value < 0.05; **p-value < 0.01; ***p-value < 0.001. CSF, cerebrospinal fluid; ESI, Eppendorf Schizophrenia Inventory; GM, grey matter; PANSS; Positive and Negative Syndrome Scale; TIV, total intracranial volume; WM, white matter.

Correlations with the ESI and the PANSS were analyzed. No significant correlations were identified between both scores and global volumes, cortical thickness, or subcortical volumes (**Figure 10**).

#### Neuropsychological findings

**Figure 11:**
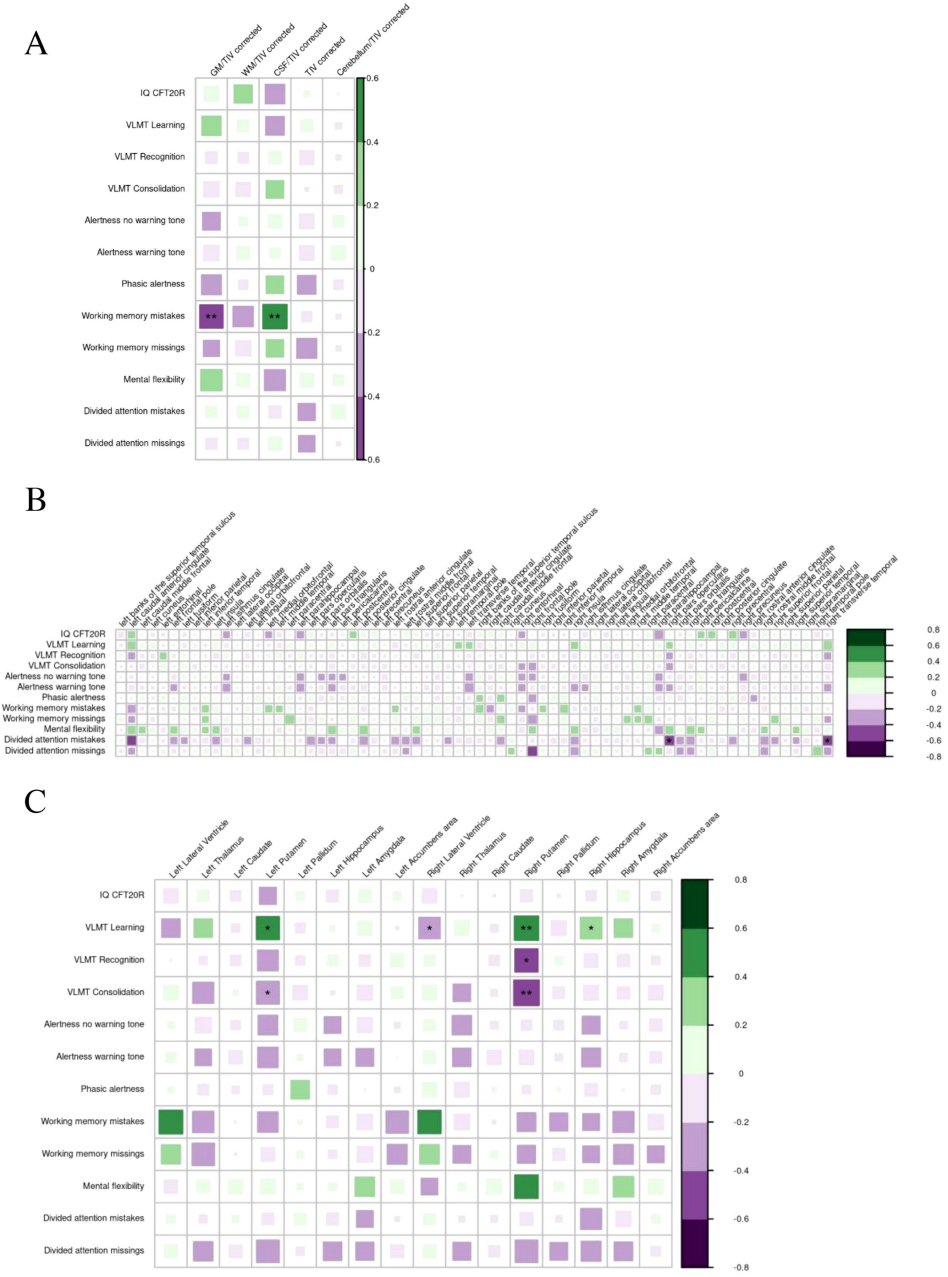
Correlation of neuropsychological findings with global volumes **(A)**, cortical thickness **(B)**, and subcortical volumes **(C)** in suspected autoimmune psychosis spectrum syndromes. The larger and more colorful the boxes are, the closer the described correlation is to significance. If an asterisk (*/**/***) appears, significance is achieved: *p-value < 0.05; **p-value < 0.01; ***p-value < 0.001. CSF, cerebrospinal fluid; GM, grey matter; IQ, intelligence quotient; TIV, total intracranial volume; WM, white matter; VLMT, Verbal Learning Memory Test; CFT20R, Culture Fair Intelligence Test 20.

Working memory mistakes in the TAP were negatively correlated with global GM volume (p<0.01) and positively with global CSF volume (p<0.01). Divided attention mistakes were negatively correlated with the cortical thickness of the right pars opercularis (p<0.05) and the right transversotemporal cortex (p<0.05). VLMT learning scores were positively correlated with the subcortical volume of the left (p<0.05) and right putamen (p<0.01) and the right hippocampus (p<0.05) and negatively correlated with the volume of the right lateral ventricle (p<0.05). The VLMT recognition score was negatively correlated with the volume of the right putamen (p<0.05) and the VLMT consolidation score was negatively correlated with the volume of the left (p<0.05) and right (p<0.01) putamen (**Figure 11**).

## Discussion

The main findings of this study indicate no relevant EEG changes but global structural GM volume reductions in patients with suspected AP spectrum syndromes. In the patient group, high IRDA/IRTA rates correlated with reduced volumes in the left caudal anterior cingulate gyrus. In addition, correlations between increases in hippocampal volume and inflammatory CSF markers were detected. Finally, the volumes of different brain areas, including the GM and right hippocampus, correlated with cognitive function but not with disease-specific scores.

***EEG*** was initially interpreted as a sensitive diagnostic tool for the detection of AP (7) as up to 90% of patients with anti-NMDA-R encephalitis showed EEG pathologies (27, 28). Accordingly, pathologies in EEG such as IRDA and IRTA were included in the diagnostic criteria for AP (2). We therefore hypothesized to detect mostly conspicuous EEG findings, but contrary to our hypothesis, this was not the case. Indeed, the results of this work suggest that at least IRDA/IRTA rates do not reliably distinguish between suspected AP spectrum syndrome patients and HCs. The reason for this could be that milder, oligosymptomatic psychiatric forms of AE were investigated in this study. Pathological EEG findings may be more frequently detectable in neurological manifestations of AE (1). The correlation analyses showed that higher IRDA/IRTA rates in the suspected AP spectrum syndrome group were associated with reduced volumes of the left caudal anterior cingulate gyrus. Anterior cingulate epilepsies were described earlier (61), so this correlation might be compatible with the electrophysiological network instabilities generated in this brain region (62, 63).

***In the global brain volume analyses***, a reduction of GM volume, an increase of CSF volume, and a trend towards reduced WM volume were identified in patients with suspected AP spectrum syndromes. The detected findings would be consistent with a global loss of volume in the sense of a reduction of brain tissue (GM and with a trend for WM) with secondary enlarged CSF spaces. MRI alterations extending beyond the limbic system have also been described for AE with well-characterized neuronal autoantibodies (31). The volume loss could be associated with the long course of the disease (31) and the late detection in many of the cases described here; however, this assumption could not be confirmed in the performed secondary analyses. Since even the acute cases did not have to have a subacute onset (<3 months), as suggested in the international consensus criteria for AP (2), these findings may nevertheless not be representative of acute AP cases (only 29% of the patients in this cohort were in an acute state).

***Focal volume comparisons*** identified a trend for a reduced cortical thickness in the left parahippocampal gyrus and the left transversotemporal gyrus, as well as an increase of the left lateral ventricle volume. The temporal cortical regions are frequent targets of CNS autoantibodies. This is indicated by the binding patterns of well-characterized neuronal autoantibodies on rat brain slices (30). However, these findings did not remain significant after correction for multiple comparisons. Increased hippocampal volumes, in contrast, were correlated with increased WBC counts in CSF. This finding could be the result of a slight autoimmune inflammatory limbic encephalitis component. Limbic encephalitis usually leads to edematous swelling of the medial temporal lobes in the initial stage (1, 64). Some of the patients in this group had antibodies (e.g., against LGI1) that can be associated with limbic encephalitis (1, 4, 5, 64). The novel antibody patterns against granule cells are also usually detected in the limbic system (as well as in the cerebellum and olfactory bulb) (65). As the disease progresses, the edema usually regresses and can even turn into atrophic changes if left untreated (55, 66, 67). Although limbic encephalitis can lead to temporal atrophy at later stages, no such atrophy was detected in this particular patient group. In addition, there were no significant differences in the WBC counts between the acute and subacute/chronic cases. Thus, the correlation appears to be associated with specific antibodies rather than the stage of the disease.

***From a clinical perspective***, these findings suggest predominantly global volume loss in the suspected AP spectrum syndrome group. Accordingly, advanced MRI analyses could support the detection of global imaging patterns in autoimmune brain processes. In addition, morphometric analyses can be used to detect hippocampal swelling and monitor the long-term course of a disease (68). However, the findings also reinforce the value of early detection and treatment, as volume loss has been associated with chronic progression in AE (17, 31). These imaging findings were correlated not with disease-specific scores (ESI or PANSS) but rather with cognitive function outcomes (on the TAP and VLMT). Thus, the alterations seem to be primarily associated not with clinical syndrome but instead with cognitive disability. In particular, a correlation was detected between low GM volume and more mistakes in working memory measurements. In addition, success in VLMT learning was positively correlated with right hippocampal volume. This finding is consistent with well-known outcomes of epilepsy research (69). Overall, these facts suggest that the morphometric findings might be clinically relevant.

***The group suspected of AP*** sp*ectrum syndromes* was clinically heterogeneous, which must be acknowledged as a major limitation of the study. Conversely, this research uniquely identified uniform underlying autoimmunity in the patient group. Patients at different disease stages and exhibiting a range of clinical presentations were included. In addition, the inclusion of patients with different CNS antibodies introduced heterogeneity. While the inclusion criteria were based on the Pollak criteria (2), a subacute onset of psychotic symptoms was not strictly required. This deviation allowed for the participation of patients with longer disease courses and broader clinical syndromes. Patients with novel CNS antibodies, whose clinical significance was still unclear, were also included. As a result, the study does not provide sufficient evidence for conclusions to be drawn about specific patient subgroups—for example, imaging findings regarding the psychiatric manifestations of NMDA-R encephalitis. However, these limitations were counterbalanced by a uniform suspected autoimmunity in all patients, which was postulated after a multimodal diagnostic work-up including EEG, CSF analysis, brain imaging, and autoantibody testing. For example, 77% of the patients had CNS autoantibodies in their CSF, which strongly suggests central autoimmunity. Patient groups with identical underlying autoimmunity are rare in psychiatry. For instance, in a systematic review of the literature on psychiatric manifestations of AE, only 145 cases were identified in the entire literature (10). Accordingly, it is currently highly difficult to monocentrically generate a uniform patient group with identical clinical syndromes, disease stages, and autoantibodies in the serum/CSF. However, our study is the first to provide overarching morphometric results for suspected AP spectrum syndrome patients.

***As further limitations***, the findings may be a consequence of long disease courses or influences of psychopharmacotherapy (albeit only in the patient group) in some cases and are therefore not generalizable to unmedicated first-episode psychosis patients. However, secondary analyses comparing patients at different disease stages—for example, acute versus nonacute or chronic—revealed no variation in the MRI group effects that were statistically significant. The inconspicuous EEG findings could also be attributed to the influence of menstrual cycle phases or to the fact that 63% of the patients had already received immunotherapies. A stratified analysis of the EEG and morphometric group comparisons based on antibody subtypes was not performed because the subgroups with identical CNS antibodies were too small. In addition, the HC group did not undergo CSF analysis or antibody testing. Thus, the results of this research—the first exploratory case-control study to apply morphometric MRI approaches to suspected AP spectrum syndromes—should be interpreted as preliminary findings that need to be replicated; they could also be used to generate further hypotheses. Future studies (ideally in multicenter settings) would benefit from larger sample sizes and more targeted approaches, such as examining patients with the same clinical syndrome and well-characterized CNS antibody at one clearly defined disease stage (e.g., the acute phase).

## Conclusion

In summary, this study employed a multimodal approach—including automated EEG, advanced morphometric MRI analysis, and CSF, antibody, and psychometric/neuropsychological testing—to provide novel insights into morphometric alterations in patients with suspected AP spectrum syndromes and these alterations’ association with cognitive, EEG, and CSF findings. Further immunopsychiatric research using advanced multimodal approaches holds strong potential to uncover specific diagnostic patterns.

## Data Availability

The raw data supporting the conclusions of this article can be made available by the authors on reasonable request.
