# Correction: Morphometric MRI findings in patients with suspected autoimmune psychosis spectrum syndromes and association with EEG slowing, CSF changes, and psychometric/neuropsychological findings

**DOI:** 10.3389/fimmu.2025.1755844

**Published:** 2026-01-13

**Authors:** Frontiers Production Office

**Affiliations:** Frontiers Media SA, Lausanne, Switzerland

**Keywords:** autoantibody, neuroinflammation, structural MRI, cortical thickness, brain

Affiliation “Universidad Popular Autónoma del Estado de Puebla, México” was erroneously given as “Facultad de Educación” for reviewer: Nayeli Huidobro.

The original version of this article has been updated.

